# Histone modification signature at myeloperoxidase and proteinase 3 in patients with anti-neutrophil cytoplasmic autoantibody-associated vasculitis

**DOI:** 10.1186/s13148-016-0251-0

**Published:** 2016-08-12

**Authors:** Jiajin Yang, Heng Ge, Caroline J. Poulton, Susan L. Hogan, Yichun Hu, Britta E. Jones, Candace D. Henderson, Elizabeth A. McInnis, William F. Pendergraft, J. Charles Jennette, Ronald J. Falk, Dominic J. Ciavatta

**Affiliations:** 1 UNC Kidney Center, Department of Medicine, Division of Nephrology and Hypertension, University of North Carolina at Chapel Hill, Chapel Hill, NC USA; 2 Department of Pathology and Laboratory Medicine, University of North Carolina at Chapel Hill, Chapel Hill, NC USA; 3 Department of Genetics, University of North Carolina at Chapel Hill, 120 Mason Farm Road, Campus Box 7264, Chapel Hill, NC 27599 USA; 4 Department of Nephrology, The Second Affiliated Hospital, School of Medicine, Xian Jiaotong University, 157 Xiwu Road, Xian, Shaanxi 710004 People’s Republic of China

**Keywords:** ANCA-associated vasculitis, Epigenetics, Autoantigens, Gene expression, Neutrophils

## Abstract

**Background:**

Anti-neutrophil cytoplasmic autoantibody (ANCA)-associated vasculitis (AAV) is a systemic autoimmune disease characterized by destructive vascular inflammation. Two prominent ANCA autoantigens are myeloperoxidase (MPO) and proteinase 3 (PR3), and transcription of *MPO* and *PRTN3*, the genes encoding the autoantigens, is associated with disease activity. We investigated whether patients with AAV have alterations in histone modifications, particularly those associated with transcriptional activation, at *MPO* and *PRTN3*.

**Results:**

We identified a network of genes regulating histone modifications that were differentially expressed in AAV patients compared to healthy controls. We focused on four genes (*EHMT1* and *EHMT2*, *ING4*, and *MSL1*) and found their expression correlated with expression of *MPO* and *PRTN3*. Methylation of histone H3K9, catalyzed by EHMT1 and EHMT2 and associated with gene silencing, was most depleted at *MPO* and *PRTN3* in patients with active disease and the highest *MPO* and *PRTN3* expression. Acetylation of histone H4K16, modified by complexes containing ING4 and MSL1 and associated with gene activation, was most enriched at *MPO* and *PRTN3* in patients with active disease and the highest *MPO* and *PRTN3* expression. Methylation at H3K4, a mark of transcriptional activation, was enriched at *MPO* and *PRTN3* in patients and healthy controls.

**Conclusions:**

*MPO* and *PRTN3* in neutrophils of AAV patients with active disease have a distinct pattern of histone modifications, which implicates epigenetic mechanisms in regulating expression of autoantigen genes and suggests that the epigenome may be involved in AAV pathogenesis.

**Electronic supplementary material:**

The online version of this article (doi:10.1186/s13148-016-0251-0) contains supplementary material, which is available to authorized users.

## Background

Anti-neutrophil cytoplasmic autoantibody (ANCA)-associated small-vessel vasculitis is an autoimmune disease characterized by autoantibodies directed against neutrophil granule proteins myeloperoxidase (MPO) or proteinase 3 (PR3). In vitro assays and in vivo mouse models have established a pathogenic role for MPO-ANCA and suggest that PR3-ANCA is pathogenic [1–4]. Despite these data, the correlation of ANCA titers and disease status is less than perfect, with reports of 25 % of patients having no relationship between clinical status and presence of ANCA [5]. In addition, naturally occurring autoantibodies to MPO in healthy individuals [6, 7] and multi-specific ANCA in ulcerative colitis [8] suggest that the characteristic vascular pathology requires additional component(s) beyond disrupted immunological tolerance to self. A prime candidate responsible for the pathology in patients with AAV is the neutrophil. Neutrophils are the major sources of PR3 and MPO, and vascular damage results from ANCA binding to these autoantigens inducing degranulation of primed neutrophils [1, 9–12]. This raises the question of whether neutrophils from healthy individuals compared to patients with AAV differ in a molecular signature, which could indicate a mechanism for neutrophil dysregulation in AAV.

A specific feature of mature peripheral blood neutrophils in patients with AAV is the presence of mRNA for *PRTN3* and *MPO* [13]. Normally, these genes are transcribed in neutrophil progenitors residing in the bone marrow [14]. To explain the observation of aberrant autoantigen expression in AAV, we proposed that peripheral blood neutrophils from patients with AAV fail to silence or maintain silencing of *PRTN3* and *MPO* due to reduced levels of the epigenetic modification histone H3 lysine 27 trimethylation (H3K27me3), associated with transcriptionally silent chromatin [15]. We hypothesize that in neutrophils of patients with AAV, a pattern of histone modifications at *PRTN3* and *MPO* genes, including histone modifications associated with transcriptional activation, is associated with disease status, but which ones are altered among the myriad possibilities is unknown.

We probed expression data from AAV patients and healthy individuals to identify differentially expressed genes that encode proteins responsible for histone modifications associated with transcriptional activity. We determined whether the levels of histone modifications at *PRTN3* and *MPO* differed between AAV patients and healthy individuals. Interestingly, measuring the levels of several histone modifications at *PRTN3* and *MPO* revealed an epigenetic signature that is related to gene expression and AAV disease status.

## Results

### Analysis of microarray expression data to identify genes that regulate chromatin modifications

**Fig. 1:**
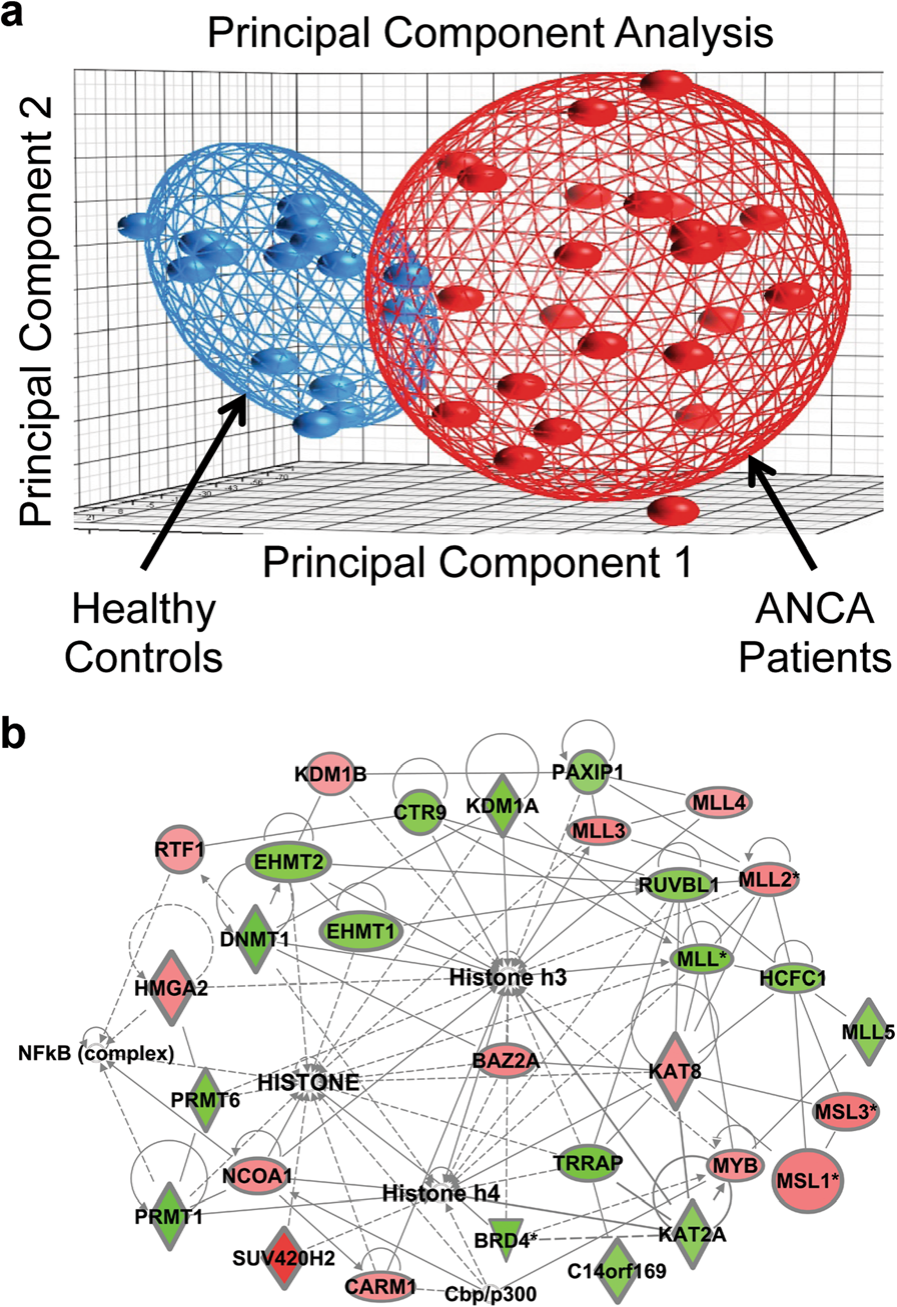
Bioinformatic analysis of microarray gene expression data comparing leukocytes from AAV patients to healthy controls. **a** Principal component analysis of the gene expression profile is shown for AAV patients, represented by *red dots*, and for healthy controls, represented by *blue dots*. **b** Gene expression data was processed with Ingenuity Pathway Analysis software to unveil biological networks. The primary network shown is for genes with functional annotations related to histone, histone H3, and/or histone H4. Genes with increased or decreased expression in AAV patients compared to healthy controls are shown in *red* and *green*, respectively

Previous studies demonstrated that *MPO* and *PRTN3* transcripts are aberrantly elevated in patients with AAV compared to healthy controls, and their expression levels are highly correlated [13]. Later, epigenetic differences were identified in patients with AAV and the transcriptional regulation of *MPO* and *PRTN3* involved epigenetic control [15]. To identify additional epigenetic mechanisms that may be faulty in patients with AAV, we analyzed microarray expression data from 25 peripheral blood leukocyte samples of AAV patients and 16 samples from healthy controls (Additional file 1: Table S1). We found 11,444 genes to be differentially regulated (≥ ± 1.2-fold and *p* < 0.05) in AAV patients. Principal component analysis (PCA) revealed the gene expression profiles in AAV patients cluster separately from expression profiles of healthy controls (Fig. 1a). The list of differentially expressed genes was filtered using Ingenuity Pathway Analysis (IPA) to identify genes associated with chromatin modifications. Among these were genes involved in controlling epigenetic marks of transcriptional activation and transcriptional silencing (Table 1). IPA also revealed that 30 of these genes were involved in a network with connections to histone, histone H3 and/or histone H4 (Fig. 1b). This discovery of differentially expressed genes responsible for regulatory histone modifications warranted further investigation of epigenetic mechanisms in AAV patients.

**Table 1:** Differentially expressed genes in patients with ANCA-associated vasculitis compared to healthy controls regulating histone post-translational modifications and DNA methylation

Gene symbol	Fragment name	Entrez gene name	ANCA/HC *p*	ANCA/HC fold change
Histone H4K16, H4K5, H4K12 acetylation				
*MSL3*	207551_s_at	male-specific lethal 3 homolog	0.0002	1.65
*MSL1*	224765_at	male-specific lethal 1 homolog	<0.0001	1.60
*KAT8*	221820_s_at	lysine acetyltransferase 8	0.0187	1.38
*BRD4*	226052_at	bromodomain containing 4	0.0003	1.38
*ING4*	218234_a	inhibitor of growth family, member 4	<0.0001	−2.05
Histone H3K4 methylation				
*MLL3*	222415_at	myeloid/lymphoid or mixed-lineage leukemia 3	<0.0001	1.53
*MLL2*	231974_at	myeloid/lymphoid or mixed-lineage leukemia 2	0.0003	1.51
*MLL4*	203419_at	myeloid/lymphoid or mixed-lineage leukemia 4	0.0107	1.26
*RTF1*	212302_at	Rtf1, Paf1/RNA polymerase II complex component	0.0409	1.24
*BCOR*	223566_s_at	BCL6 corepressor	<0.0001	−1.74
*KDM1A*	212348_s_at	lysine-specific demethylase 1A	0.0002	−1.57
*C14orf169*	219526_at	lysine-specific demethylase NO66	0.0002	−1.34
Histone H3K14 acetylation				
*BRD4*	226052_at	bromodomain containing 4	0.0003	1.38
Histone H3K36 methylation				
*NSD1*	225654_at	nuclear receptor binding SET domain protein 1	0.0208	1.34
*BCOR*	223566_s_at	BCL6 corepressor	<0.0001	−1.74
*C14orf169*	219526_at	lysine-specific demethylase NO66	0.0002	−1.34
Histone H3K9 methylation				
*EHMT1*	222873_s_at	euchromatic histone-lysine N-methyltransferase 1	0.0084	−1.41
*EHMT2*	202326_at	euchromatic histone-lysine N-methyltransferase 2	0.0013	−1.44
*ARID4B*	212591_at	AT rich interactive domain 4B (RBP1-like)	<0.0001	−1.51
Histone H4K20 methylation				
*ARID4B*	212591_a	AT rich interactive domain 4B (RBP1-like)	<0.0001	−1.51
DNA methylation				
*DNMT1*	201697_s_at	DNA (cytosine-5-)-methyltransferase 1	<0.0001	−2.03

### Expression of histone modifying genes

From our filtered list of differentially expressed genes, we evaluated two genes (Euchromatic histone-lysine *N*-methyltransferase 1 and 2, *EHMT1/GLP* and *EHMT2/G9a*) that regulate histone H3K9 methylation, associated with transcriptionally silent chromatin, and two genes (male sex lethal 1 homolog, *MSL1* and insulin growth factor, *ING4*) that regulate histone acetylation, associated with transcriptionally active chromatin. We chose *EHMT1/GLP* and *EHMT2/G9a* to address whether the H3K9me2 pathway was involved in epigenetic silencing of *MPO* and *PRTN3* expression. We investigated genes that regulate histone acetylation as a mechanistic explanation for increased expression of *MPO* and *PRTN3* in patients with AAV. MSL1 is a subunit of a human acetyltransferase (HAT) complex that acetylates histone H4K16 [16]. ING4 plays a complex role in gene regulation [17]. It associates with HBO1 HAT complex [18], but ING4 can also interact with NFkB and enhance HDAC-1 levels at promoters [19]. Microarray analysis revealed expression of *EHMT1/GLP*, and *EHMT2/G9a* was significantly depleted in leukocytes from AAV patients compared to healthy controls, while expression of *MSL1* was statistically elevated (Table 1). Interestingly, leukocytes from AAV patients had reduced *ING4* expression compared to healthy controls. Expression levels of *EHMT2/G9a* and *ING4* negatively and *MSL1* positively correlated with *PRTN3* and *MPO* mRNA levels (Table 2). *EHMT1/GLP* did not show a significant correlation with *PRTN3* and *MPO* mRNA.

**Table 2:** Correlation of expression levels of *PRTN3* and *MPO* with genes associated with histone modifications

	Microarray		Quantitative RT-PCR	
Gene name	Correlation with *PRTN3* expression	Correlation with *MPO* expression	Correlation with *PRTN3* expression	Correlation with *MPO* expression
*MPO*	*r* = 0.840 *p* < 0.0001		*r* = 0.920 *p* < 0.0001	
*EHMT1*	*r* = −0.213 *p* = 0.182	*r* = −0.148 *p* = 0.357	*r* = −0.436 *p* < 0.0001	*r* = −0.391 *p* < 0.0001
*EHMT2*	*r* = −0.461 *p* = 0.0024	*r* = −0.537 *p* = 0.0003	*r* = −0.396 *p* < 0.0001	*r* = −0.400 *p* < 0.0001
*MSL1*	*r* = −0.463 *p* = 0.0006	*r* = −0.412 *p* = 0.0046	*r* = −0.469 *p* < 0.0001	*r* = −0.409 *p* < 0.0001
*ING4*	*r* = −0.515 *p* = 0.0023	*r* = −0.433 *p* = 0.0075	*r* = −0.516 *p* < 0.0001	*r* = −0.477 *p* < 0.0001

**Fig. 2:**
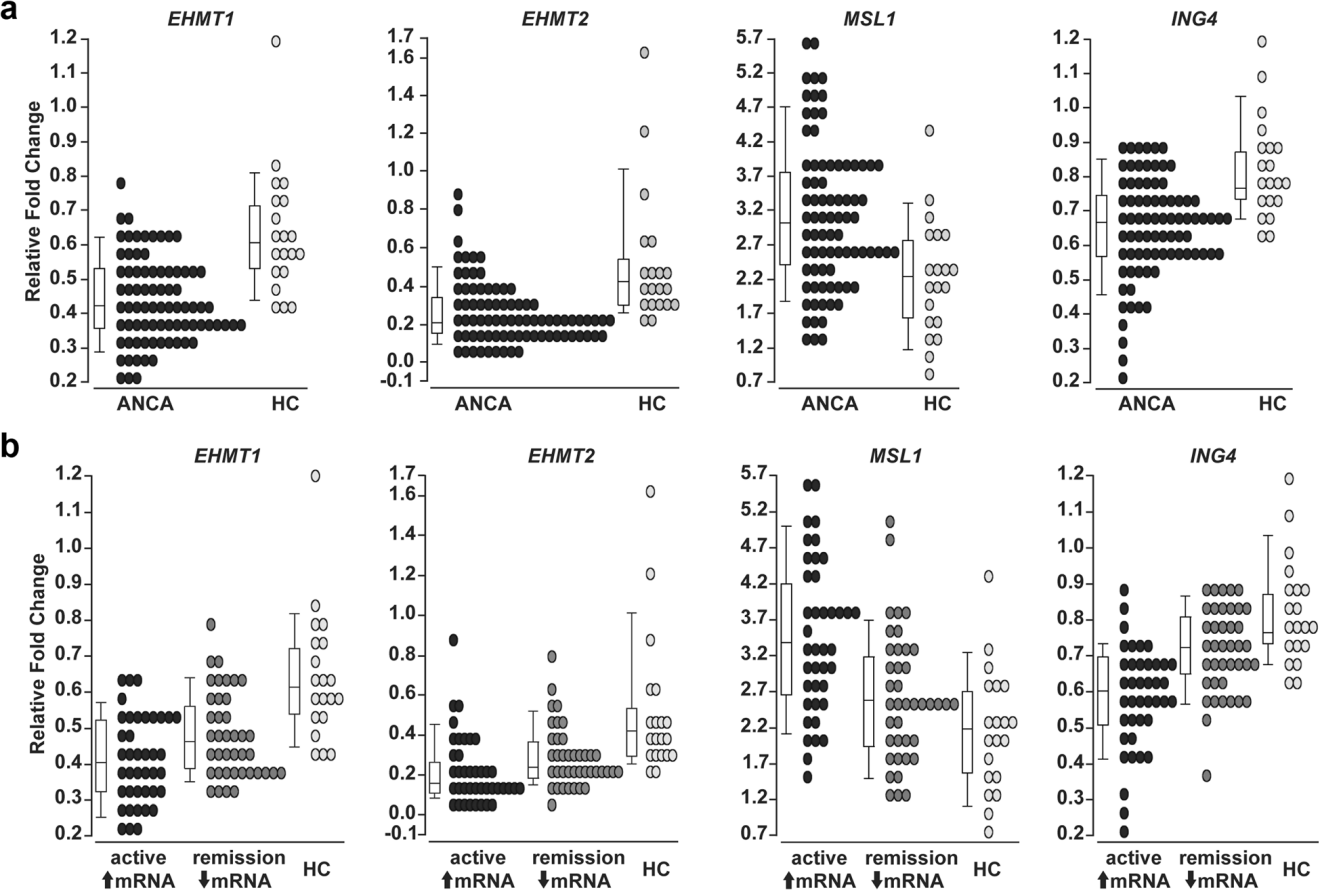
Quantitative RT-PCR analysis of candidate genes in leukocytes from AAV patients and health controls. **a** Quantitative RT-PCR was performed on RNA isolated from total leukocytes of AAV patients (*n* = 80, *black ovals*) and healthy controls (HC; *n* = 20, *light gray ovals*). Expression is reported as a relative fold change for *EHMT1* (ANCA 0.51 ± 0.12 versus HC 0.69 ± 0.17, *p* < 0.0001), *EHMT2* (ANCA 0.26 ± 0.17 versus HC 0.51 ± 0.35, *p* < 0.0001), *MSL1* (ANCA 3.07 ± 1.07 versus HC 2.16 ± 0.89, *p* = 0.0009), and *ING4* (ANCA 0.63 ± 0.14 versus HC 0.79 ± 0.14, *p* < 0.0001). **b** AAV patients were divided into two groups: (1) patients with active disease (BVAS ≥ 3) and high expression of autoantigen genes *PRTN3* and *MPO* (↑mRNA, *black ovals*, *n* = 40), (2) patients in remission (BVAS = 0) and low expression of autoantigen genes *PRTN3* and *MPO* (↓mRNA, *gray ovals*, *n* = 40). Expression comparing AAV patients with active disease and AAV patients in remission is reported as a relative fold change for *EHMT1* (↑mRNA 0.47 ± 0.11 versus ↓mRNA 0.54 ± 0.11, *p* = 0.0172), *EHMT2* (↑mRNA 0.22 ± 0.17 versus ↓mRNA 0.29 ± 0.15, *p* = 0.0074), *MSL1* (↑mRNA 3.49 ± 1.07 versus ↓mRNA 2.64 ± 0.91, *p* = 0.0004), and *ING4* (↑mRNA 0.57 ± 0.14 versus ↓mRNA 0.69 ± 0.11, *p* < 0.0001). (Note: the median expression value is represented by the *line in the box* of the box and whisker plot, while mean ± standard deviation is listed in figure legend)

Quantitative RT-PCR (qRT-PCR), to confirm the microarray results, was used to measure mRNA levels for *MPO*, *PRTN3*, *EHMT1* and *2*, *MSL1*, and *ING4* in total leukocytes from a separate cohort of 20 healthy controls and 80 AAV patients (Additional file 2: Table S2). The patients were divided evenly into MPO-ANCA and PR3-ANCA serotypes, and each ANCA serotype was divided into 20 remission patients (BVAS = 0) and 20 active patients (BVAS ≥ 3). Quantitative RT-PCR revealed that *EHMT1*, *EHMT2*, and *ING4* were statistically reduced in leukocytes from AAV patients (*n* = 80) compared to healthy controls (*n* = 20). *MSL1* was statistically elevated in AAV patients (Fig. 2a). As observed previously, in this set of patient samples, expression of *MPO* and *PRTN3* was highly correlated. Expression levels of *EHMT1*, *EHMT2*, and *ING4* negatively and *MSL1* positively correlated with *PRTN3* (ANCA 351.38 ± 608.75 versus HC 10.01 ± 8.98, *p* < 0.0001) and *MPO* (ANCA 643.41 ± 1106.44 versus HC 36.83 ± 18.27, *p* < 0.0001) mRNA levels (Table 2). These data suggest an association exists between genes that regulate histone modifications linked to transcriptional status and the expression *MPO* and *PRTN3*.

This association is further supported when comparing the expression level of *EHMT1* and *2*, *ING4*, and *MSL1* in healthy controls to AAV patients with active disease (BVAS ≥ 3) and high *MPO* and *PRTN3* mRNA (two standard deviations above the mean for healthy controls), and AAV patients in remission (BVAS = 0) with low *MPO* and *PRTN3* mRNA (no different from healthy controls). Compared to healthy controls, the expression of *EHMT1* and *2*, *ING4*, and *MSL1* changes more dramatically in AAV patients with active disease and elevated *MPO* and *PRTN3* mRNA than in AAV patients in remission with low *MPO* and *PRTN3* mRNA (Fig. 2b). These relationships suggest that expression of genes that regulate histone modifications is associated with disease activity.

A recent report describes a link between steroid therapy and expression changes of autoantigen genes [20]; therefore, it is conceivable that the expression differences of histone modifying genes are a response to therapy. However, in the cohort of patients used to measure expression by qRT-PCR, there is no significant difference in the expression of the four histone-modifying genes, *EHMNT1*, *EHMT2*, *ING4*, *MSL1*, in patients in remission and receiving corticosteroids and patients in remission receiving another therapy, or between patients in remission receiving no therapy and patients receiving another therapy. Only the expression of *MSL1* is significantly different (increased) in remitting patients on corticosteroids compared to remitting patients receiving no therapy (Additional files 2 and 3: Table S2 and Figure S1). This suggests that corticosteroid treatment alone does not explain changes in gene expression of histone-modifying genes.

**Fig. 3:**
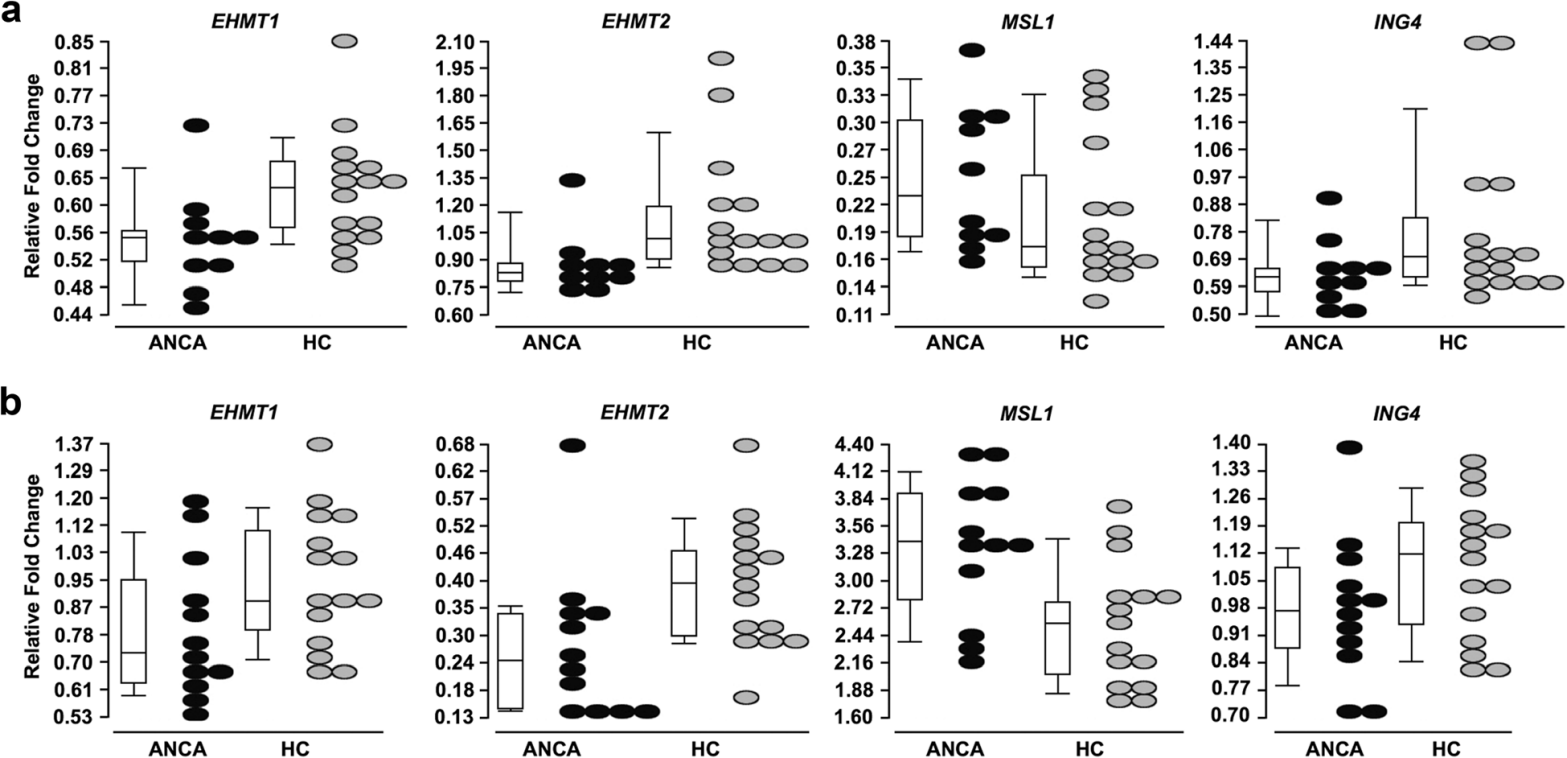
Quantitative RT-PCR analysis of candidate genes in purified monocytes and neutrophils from AAV patients and health controls. **a** Quantitative RT-PCR was performed on RNA isolated from monocytes of AAV patients (*n* = 10, *black ovals*) and healthy controls (HC; *n* = 15, *light gray ovals*). Expression is reported as a relative fold change for *EHMT1* (ANCA 0.55 ± 0.08 versus HC 0.63 ± 0.09, *p* = 0.0213), *EHMT2* (ANCA 0.88 ± 0.19 versus HC 1.15 ± 0.35, *p* = 0.0043), *MSL1* (ANCA 0.21 ± 0.07 versus HC 0.25 ± 0.07, *p* = 0.192), and *ING4* (ANCA 0.64 ± 0.12 versus HC 0.80 ± 0.29, *p* = 0.102). **b** Quantitative RT-PCR was performed on RNA isolated from neutrophils of AAV patients (*n* = 12, *black ovals*) and healthy controls (HC; *n* = 15, *light gray ovals*). Expression is reported as a relative fold change for *EHMT1* (ANCA 0.80 ± 0.22 versus HC 0.95 ± 0.21, *p* = 0.0481), *EHMT2* (ANCA 0.28 ± 0.16 versus HC 0.40 ± 0.13, *p* = 0.0043), *MSL1* (ANCA 3.35 ± 0.74 versus HC 2.55 ± 0.63, *p* = 0.0157), and *ING4* (ANCA 0.98 ± 0.19 versus HC 1.08 ± 0.18, *p* = 0.180). (Note: the median expression value is represented by the *line in the box* of the box and whisker plot, while mean ± standard deviation is listed in figure legend)

The altered expression of the four histone-modifying genes detected in total leukocytes raises the question of which cell types in the peripheral blood of patients with AAV are responsible for the differential gene expression. To test this, we isolated RNA from purified monocytes and neutrophils and performed qRT-PCR to measure expression of *EHMT1*, *EHMT2*, *ING4*, and *MSL1*. In general, patients with AAV expressed less *EHMT1* and *EHMT2* compared to healthy controls in both monocytes and neutrophils (Fig. 3a, b, respectively). *MSL1* mRNA was significantly elevated in neutrophils (Fig. 3b) from patients compared to healthy controls, but was not significantly elevated in monocytes (Fig. 3a). Although the level of *ING4* message in monocytes and neutrophils tended to be lower in patients than healthy controls, the difference was not significant. These results point to both monocytes and neutrophils as sources of the reduced expression of *EHMT1* and *EHMT2*, while the increased expression of *MSL1* in patients appears to occur predominately in neutrophils.

### Reduced levels of histone mark regulating transcriptional repression in patients with ANCA-associated vasculitis

**Fig. 4:**
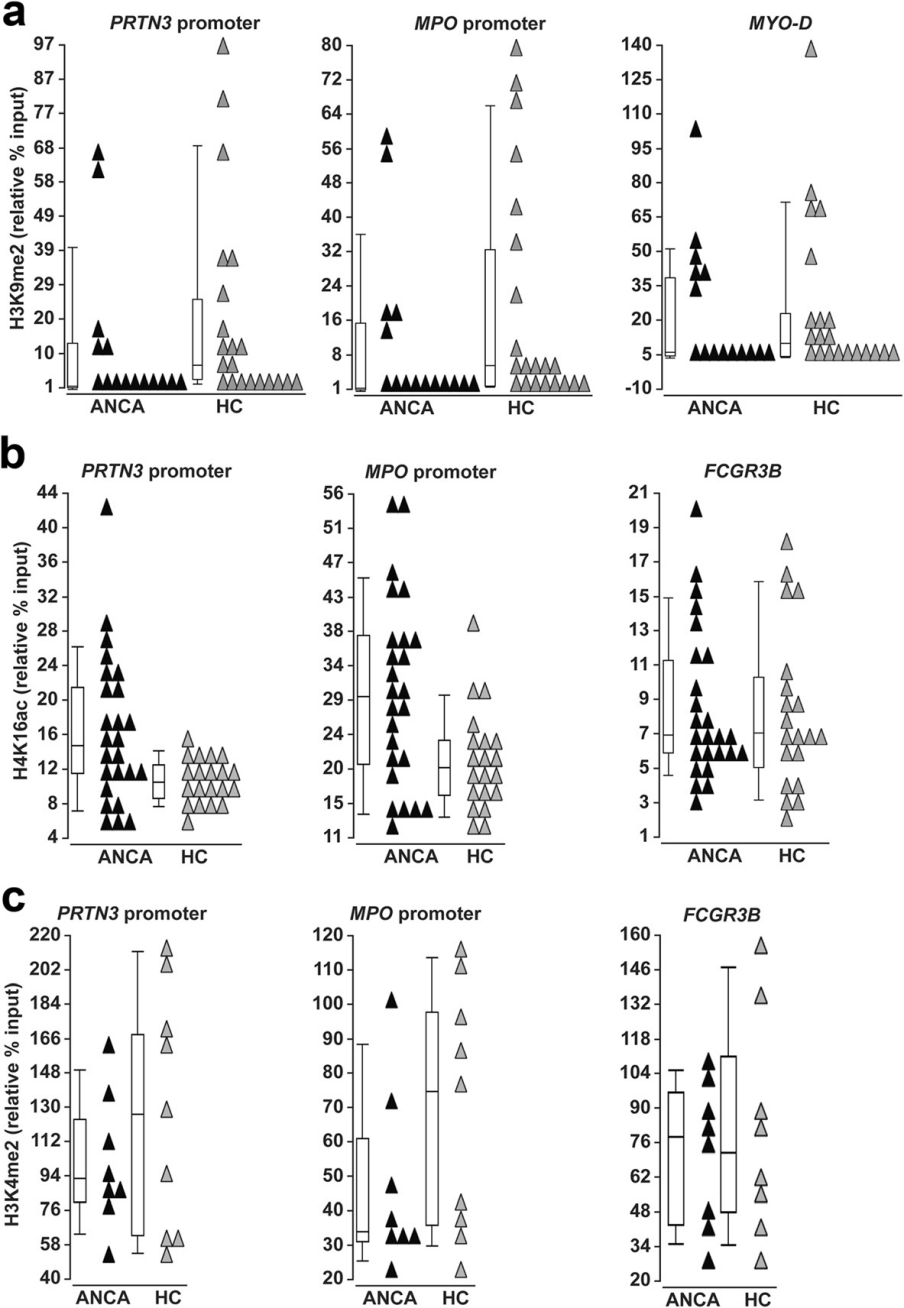
Chromatin immunoprecipitation (ChIP)-quantitative PCR analysis for histone modifications at autoantigen genes in AAV patients and healthy controls. **a** ChIP-qPCR was performed on AAV patients (ANCA, *black triangles*, *n* = 15) and healthy controls (HC; *gray triangles*, *n* = 21) for H3K9me2. The level of the H3K9me2 modification is reported as relative percent of input at the *PRTN3* promoter (ANCA 12.44 ± 22.10, HC 21.17 ± 28.55; *p* = 0.0192), *MPO* promoter (ANCA 11.37 ± 19.01, HC 20.01 ± 28.55; *p* = 0.0247), and a control gene, *MYO-D* (ANCA 21.42 ± 28.78, HC 23.63 ± 35.53; *p* = 0.702). **b** ChIP-qPCR for H4K16ac was performed on AAV patients (ANCA, *black triangles*, *n* = 25) and healthy controls (HC; *gray triangles*, *n* = 20). The level of the H4K16ac modification is reported as relative percent of input at the *PRTN3* promoter (ANCA 17.10 ± 9.14, HC 10.83 ± 2.73; *p* = 0.0116), *MPO* promoter (ANCA 30.30 ± 12.89, HC 21.06 ± 6.67; *p* = 0.0132) and at a control gene, *FCGR3B* (ANCA 9.19 ± 4.74, HC 8.83 ± 5.14, *p* = 0.864). **c** ChIP-qPCR for H3K4me2 was performed on AAV patients (ANCA, *black triangles*, *n* = 8) and healthy controls (HC, *gray triangles*, *n* = 8). The level of the H3K4me2 modification is reported as relative percent of input at the *PRTN3* promoter (ANCA 50.68 ± 18.15, HC 63.78 ± 32.86, *p* = 0.532), *MPO* promoter (ANCA 24.17 ± 14.80, HC 36.09 ± 18.88, *p* = 0.136), and at a control gene, *FCGR3B* (ANCA 74.01 ± 30.26, HC 82.74 ± 46.52, *p* = 0.878) (Note: the level of the indicated histone modification was calculated using raw Ct values from qPCR of diluted input sample. The median expression value is represented by the *line in the box* of the box and whisker plot, while mean ± standard deviation is listed in figure legend)

**Fig. 5:**
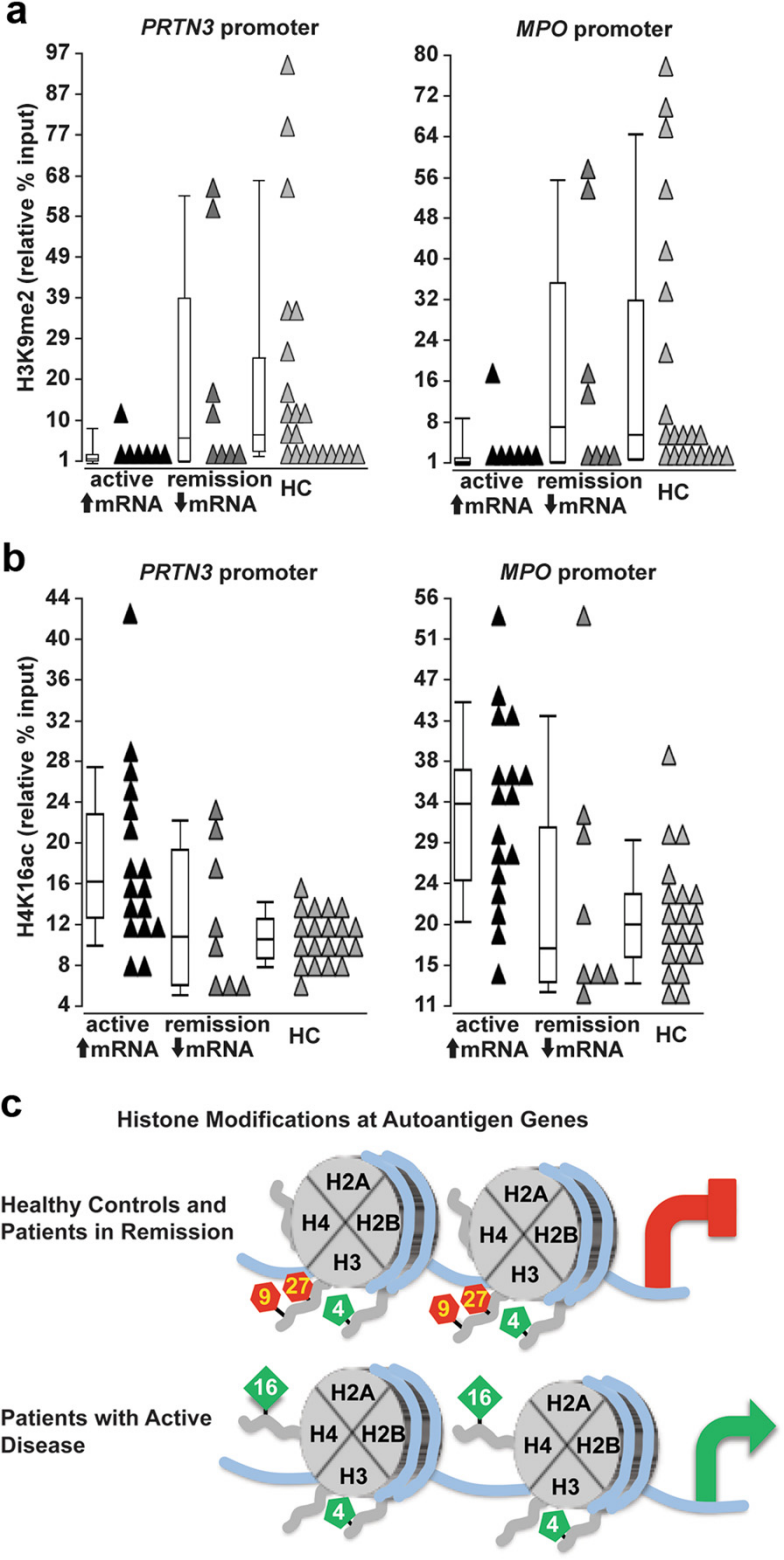
ChIP-quantitative PCR analysis for histone modifications at autoantigen genes in ANCA-patients with high and low *PRTN3* and *MPO* expression. **a** The level of H3K9me2 is reported as relative percent input for AAV patients with active disease (BVAS ≥ 3) and high expression of *PRTN3* and *MPO* (↑mRNA, *black triangles*, *n* = 7) and for healthy controls (HC; *light gray triangles*, *n* = 21) at the *PRTN3* promoter (↑mRNA 3.41 ± 5.20, HC 21.17 ± 28.55; *p* = 0.0068) and the *MPO* promoter (↑mRNA 3.34 ± 6.02, HC 20.01 ± 26.00; *p* = 0.0058). The level of H3K9me2 is reported as relative percent input for AAV patients in remission (BVAS = 0) and low expression of *PRTN3* and *MPO* (↓mRNA, *gray triangles*, *n* = 8) for healthy controls at the *PRTN3* promoter (↓mRNA 20.33 ± 28.30, HC 21.17 ± 28.55; *p* = 0.294) and at the *MPO* promoter (↓mRNA 18.41 ± 23.88, HC 20.01 ± 26.00; *p* = 0.393). **b** The level of H4K16ac is reported as relative percent input for AAV patients with active disease (BVAS ≥ 3) and high expression of *PRTN3* and *MPO* (↑mRNA, *black triangles*, *n* = 17) and for healthy controls (HC; *light gray triangles*, *n* = 20) at the *PRTN3* promoter (↑mRNA 19.12 ± 9.31, HC 10.83 ± 2.73; *p* = 0.0009) and the *MPO* promoter (↑mRNA 33.26 ± 11.03, HC 21.56 ± 6.67; *p* = 0.0006). The level of H4K16ac is reported as relative percent input for AAV patients in remission (BVAS = 0) and low expression of *PRTN3* and *MPO* (↓mRNA, *gray triangles*, *n* = 8) for healthy controls at the *PRTN3* promoter (↓mRNA 12.81 ± 7.55, HC 10.83 ± 2.73; *p* = 0.939) and at the *MPO* promoter (↓mRNA 24.02 ± 15.02, HC 21.56 ± 6.67; *p* = 0.859). (Note: the level of the indicated histone modification was calculated using raw Ct values from qPCR of diluted input sample. The median expression value is represented by the *line in the box* of the box and whisker plot, while mean ± standard deviation is listed in figure legend.) **c** Illustration depicts a model for modifications of histone tails at promoters of *PRTN3* and *MPO* in healthy controls and AAV patients in remission (*top*), and in AAV patients with active disease (*bottom*)

Since we found decreased expression of *EHMT1* and *2*, we tested whether there is a reduction in H3K9me2 at *MPO* and *PRTN3* genes. Chromatin immunoprecipitation (ChIP) was performed on neutrophils isolated from 15 AAV patients and 21 healthy controls (Additional file 4: Table S3). We detected a modest but significant decrease in the level of H3K9me2 at both the *MPO* and *PRTN3* gene promoters in AAV patients compared to healthy controls (Fig. 4a). At a transcriptionally silent gene in neutrophils, *MYO-D*, there was a similar enrichment for H3K9me2 between AAV patients and healthy controls. As described for the expression of *EHMT1* and *2* compared to *MPO* and *PRTN3* expression, H3K9me2 was more dramatically depleted at *PRTN3* and *MPO* promoter in AAV patients with active disease and high *MPO* and *PRTN3* mRNA compared to controls, but in AAV patients in remission with low *MPO* and *PRTN3* mRNA, H3K9me2 does not differ from the healthy controls (Fig. 5a). We detected a modest but significant negative correlation between H3K9me2 levels at *PRTN3* and *MPO* promoters and expression of *MPO* and *PRTN3* (*r* = −0.346, *p* = 0.0386 *MPO* prm and *MPO* expression; *r* = −0.373, *p* = 0.0252 *PRTN3* prm and *PRTN3* expression) (Table 3). This inverse relationship is consistent with a role for this modification in silencing *MPO* and *PRTN3* in mature neutrophils, and the H3K9me2 silencing pathway may be disrupted in AAV patients.

**Table 3:** Correlation of *PRTN3* and *MPO* expression with H3K9me2 and H4K16ac levels at *PRTN3* and *MPO* promoter

	H3K9me2 (*N* = 36)		H4K16ac (*N* = 40)	
Gene name	Correlation with *PRTN3* expression	Correlation with *MPO* expression	Correlation with *PRTN3* expression	Correlation with *MPO* expression
*PRTN3* promoter	*r* = −0.373 *p* = 0.0252		*r* = −0.303 *p* = 0.0563	
*MPO* promoter		*r* = −0.346 *p* = 0.0386		*r* = −0.427 *p* = 0.0058

### Elevated levels of histone modifications regulating transcriptional activation in patients with ANCA-associated vasculitis

In light of our expression data, we wondered if marks coincident with transcriptional activation were elevated in AAV patients. Because of increased expression of *MSL1* (encoding a histone H4 HAT) in AAV patients, we used ChIP to examine the level of H4K16 acetylation (H4K16ac) at *MPO* and *PRTN3* promoters. H4K16ac was significantly elevated in patients (*n* = 25) compared to healthy controls (*n* = 20), but not different between patients and controls at the *FCGR3B* gene (Fig. 4b). The level of H4K16ac at the *PRTN3* promoter showed a modest correlation with *PRTN3* expression (*r* = 0.303, *p* = 0.0563), and H4K16ac at the *MPO* promoter showed a modest, significant correlation with *MPO* expression (*r* = 0.427, *p* = 0.0058) (Table 3). Similar to the relationship described above for the level of H3K9me2 and *MPO* and *PRTN3* expression, the level of H4K16ac was markedly higher in AAV patients with active disease and high *MPO* and *PRTN3* expression versus patients in remission with low *MPO* and *PRTN3* expression (Fig. 5b).

Because genes involved in H3 acetylation were increased in AAV patients (Table 1), we performed ChIP for H3K9,14ac. Low levels of this modification were detected at *PRTN3* and *MPO* promoters and not different between AAV patients and healthy controls (Additional file 5: Figure S2a). This was surprising given that preliminary data that indicated H3K9,14ac levels were closely associated with expression of *MPO* and *PRTN3* in HL60 cells (Additional file 5: Figure S2b); however, this underscores the idea that regulatory mechanisms for the same genes may differ in cell lines compared to primary human cells.

Several mixed-lineage leukemia genes (*MLL2*, *3*, and *4*) were expressed more in AAV patients compared to healthy controls. Since the MLLs regulate transcriptional activation and catalyze methylation of H3K4, we measured H3K4me2 levels. ChIP for H3K4me2 revealed that *PRTN3* and *MPO* promoters were highly enriched for this modification in both AAV patients (*n* = 8) and healthy controls (*n* = 9) (Fig. 4c). Because the promoters of *MPO* and *PRTN3* in healthy controls have high levels (slightly higher than the promoters in ANCA patients) of H3K4me2, these genes may be in a transcriptionally competent state, ready to respond at the transcriptional level to stimuli, but are maintained silent by repressive histone modifications. Figure 5c summarizes the ChIP data for three different histone modifications and illustrates an epigenetic signature at *MPO* and *PRTN3* genes related to disease status and autoantigen expression.

## Discussion

### Differential expression of genes encoding histone-modifying enzymes

We explored whether patients with AAV had changes in expression of genes encoding factors that establish and maintain chromatin modifications. Analysis of microarray data comparing AAV patients to healthy controls revealed a network of genes related to chromatin modifications. Quantitative RT-PCR confirmed differential expression of *EHMT1/GLP* and *EHMT2/G9a*, encoding H3K9 methyltransferases, and *MSL1* and *ING4*, encoding H4K16 acetyltransferases. The expression of these four genes correlated with expression of *MPO* and *PRTN3*, and the expression of *MPO* and *PRTN3* is strongly correlated. Furthermore, expression of *EHMT1* and *2*, *MSL1* and *ING4* differed more between healthy controls and AAV patients with active disease (BVAS ≥ 3) and the highest levels of *MPO* and *PRTN3* mRNA than between healthy controls and AAV patients in remission (BVAS = 0) with low levels of *MPO* and *PRTN3* mRNA.

### Histone modifications at autoantigen genes associated with disease activity

We also explored in patients with AAV whether the histone modifications regulated by these differentially expressed genes were altered at *MPO* and *PRTN3* genes. In neutrophils from patients with high *MPO* and *PRTN3* mRNA and active disease, the *MPO* and *PRTN3* promoters were depleted for H3K9me2 and enriched for H4K16ac compared to healthy controls and patients in remission. In contrast, another histone modification that is associated with gene activation showed similar levels between AAV patients and healthy controls (H3K9,14ac), while H3K4me2 levels were enriched at *MPO* and *PRTN3* in patients and healthy controls. These data and our previous ChIP studies on neutrophils demonstrate that *MPO* and *PRTN3* genes in healthy controls and AAV patients in remission are enriched for silencing marks H3K27me3 and H3K9me2 and the activation mark H3K4me2. In contrast, *MPO* and *PRTN3* genes in active AAV patients have reduced levels of H3K27me3 and H3K9me2, maintain enrichment of H3K4me2, and gain the activation mark H4K16ac.

Two results from the expression analysis were unexpected. First, we did not identify changes in gene expression in AAV patients for genes encoding Polycomb Repressor Complex-2 (PRC2) subunits. However, the absence of changes in PRC2 genes is consistent with a model we proposed in which the deficit in H3K27me3 genes results from a failure to recruit PRC2 to *MPO* and *PRTN3* via RUNX3, or histone demethylase activity [15]. Second, although our initial measurements of H3K9me2 showed a modest decrease in AAV patients, we found reduced expression of *EHMT1/GLP* and *EHMT/G9a* in patients. Re-examining the ChIP data for H3K9me2 revealed the modification was more dramatically depleted at *MPO* and *PRTN3* in active AAV patients with high autoantigen gene expression. This suggests that in addition to H3K27me3, H3K9me2 and the factors that catalyze this histone mark contribute to the transcriptional silencing of ANCA autoantigen genes in healthy individuals and AAV patients in remission.

### H3K27me3 and H3K4me2 enriched at autoantigen genes in PMNs from healthy individuals and AAV patients in remission

The levels of both H3K27me3 and H3K4me2 at *MPO* and *PRTN3* genes raises the intriguing possibility that in healthy controls and remitting patients, these genes are maintained in a bivalent epigenetic state. A model to explain the function of this chromatin state proposes that bivalent genes are suppressed in pluripotent cells but poised for activation later in development [21, 22]. Genome-wide chromatin studies have detected bivalent domains in differentiated cells, particularly of the hematopoietic lineage [23, 24]. An exciting possibility is that in peripheral blood neutrophils, the *PRTN3* and *MPO* genes adopt a bivalent mode of transcriptional control and exist as transcriptionally poised genes. Although a poised chromatin state is consistent with studies demonstrating inducible transcriptional activity in neutrophils [25–29], it is possible that the apparent bivalent signature represents a mosaic between neutrophils with H3K27me3 and neutrophils with H3K4me2. If a population of neutrophils contains a mixture of cells with either one of these two histone modifications, it would suggest that neutrophils with H3K4me2 employ another silencing mechanism; distinguishing these possibilities warrants further investigation.

### Other potential cell types responsible for differential expression of autoantigen genes

The model of epigenetic control we propose assumes that the elevated expression of *MPO* and *PRTN3* in AAV is primarily from neutrophils. Others have reported increased *MPO* and *PRTN3* expression in monocytes, myeloid progenitors, or low density granulocytes [30–32]. This could explain the moderate correlations we observe between *MPO* and *PRTN3* expression from total leukocytes and histone modifications at *MPO* and *PRTN3* in purified neutrophils. Evidence from in situ hybridization [13] and comparison of expression with differential blood counts (manuscript in preparation) indicates AAV patients have increased expression of *MPO* and *PRTN3* in peripheral neutrophils, but this does not exclude the contribution of other cell types to *MPO* and *PRTN3* expression. Indeed, the differential expression of chromatin-modifying factors was identified in total leukocytes; therefore, it is possible that altered expression of these factors in patients with AAV could occur in other peripheral blood cells. We identified differential expression of *EHMT1*, *EHMT2*, and *MSL1* in neutrophils, but we also detected reduced levels of *EHMT1* and *EHMT2* in monocytes. The increased expression of *MSL1* in neutrophils of patients with AAV is consistent with the elevated levels of H4K16ac we detected at *MPO* and *PRTN3* in neutrophils from patients with active disease. Intriguingly, monocytes, which did not differentially expression *MSL1* between patients and healthy controls, may regulate transcriptional activation through a different network of epigenetic regulators. Low density granulocytes could also have altered expression of these histone-modifying genes.

By extension, the pattern of histone modifications we identified in neutrophils at *MPO* and *PRTN3* might occur in other cell types. In fact, publically available data from the Encyclopedia of DNA Elements (ENCODE) project corroborates enrichment of H3K4me2 at the promoter and within the gene body of *PRTN3* and *MPO*. Conversely, very little enrichment of H3K27me3 at *MPO* and *PRTN3* is reported for monocytes. Depending on the cell type, the transcriptional output of these autoantigen genes in patients with active AAV may use similar but not identical chromatin regulatory mechanisms. Investigating epigenetic patterns of other myeloid lineages would further define transcriptional regulatory mechanisms during AAV.

### Heterogenous surface PR3 expression among neutrophils

We report the level of specific histone modifications at autoantigen genes in samples of total circulating neutrophils; however, several reports have documented a subset of neutrophils with membrane bound PR3 (mPR3) and the surface glycoprotein CD177. The fraction of neutrophils positive for mPR3 and surface CD177 ranges from 0 to 100 %, is genetically determined and stable, and is elevated in patients with AAV [33–36]. It is conceivable that this subset of neutrophils could influence the level of a particular histone modification measured at autoantigen genes. For instance, the observation that histone modifications are more dramatically altered in patients with increased expression might also reflect patients with a larger percentage of mPR3 and CD177 positive neutrophils. However, separate reports have described conflicting results on whether mPR3 and CD177 positive neutrophils have elevated *PRTN3* expression [37, 38]; thus, the association of a histone modification signature with increased autoantigen expression may not depend on mPR3 and surface CD177 positivity. In addition, PR3-ANCA has been shown to stimulate neutrophil activation regardless of mPR3 status [39]. This is consistent with the hypothesis that dysregulation of neutrophil transcriptional status, likely the result of epigenetic changes, predisposes neutrophils to activation.

### Limitations of current epigenetic analysis, and rationale for future epigenomic studies in patients with AAV

Although the role epigenetic mechanisms play in AAV and other vasculitides is being investigated more intensively [40], challenges still remain. For instance, this study does not distinguish whether or not epigenetic changes are a consequence of AAV or are an integral component of AAV pathogenesis, and our patient samples prevented an investigation of the role of therapy on the chromatin status. However, our results provide a rationale for further investigating the role of epigenetic changes in AAV. Measuring the status of histone modifications longitudinally will more accurately assess the relationship of a histone signature with disease status. Genome-wide mapping of histone modifications will define chromosomal domains and genes within those domains with shared regulatory mechanisms. This is critical to identify potential chromatin regulatory networks that play a role in neutrophil activation in AAV. Coupled with data from genome-wide association studies epigenomic profiling in AAV, especially expanded to include other cell types, will inform potential function of genetic variants [41].

## Conclusions

While additional questions remain to be answered, the close association of specific histone modifications with active AAV is consistent with the hypothesis that an epigenetic signature corresponds to disease status, and suggests that a dynamic epigenome is involved in the pathogenesis of AAV. Thus, in addition to the presence of autoantibodies, the plasticity of the epigenome may promote the development of AAV.

## Methods

### Patients

Patients with biopsy-proven anti-neutrophil cytoplasmic autoantibody (ANCA) systemic vasculitis or renal-limited disease enrolled in this study were diagnosed between 1985 and 2013 and followed in the Glomerular Disease Collaborative Network (GDCN) [42, 43]. All study materials were given Institutional Review Board approval for human subjects (IRB study #97-0523) by the University of North Carolina Office of Human Research Ethics. Study subjects gave informed, written consent and participated according to UNC IRB guidelines. A total of 122 patients with ANCA vasculitis and 71 healthy controls were included in this study. Patients were diagnosed according to the Chapel Hill Consensus Conference [44, 45]. ANCA serotypes were determined by indirect immunofluorescence and/or antigen-specific proteinase (PR3) and myeloperoxidase (MPO) enzyme-linked immune-absorbent assays (ELISA) [46]. Disease activity was determined using the 2003 Birmingham Vasculitis Activity Score (BVAS) [47]. Remission was defined as BVAS = 0 and no clinical or laboratory evidence of active disease, and active disease was defined as a BVAS ≥ 3 with clinical and/or laboratory evidence of disease. Among the ANCA cohort, 25 samples from 21 patients were enrolled in a microarray study listed in Additional file 1: Table S1, 80 samples from 80 patients in a quantitative PCR study of total leukocytes listed in Additional file 2: Table S2, 22 samples (10 monocytes and 12 neutrophils) from 13 patients in a quantitative PCR study of purified cell types listed in Additional file 2: Table S2 (samples TM81-TM93), and 32 samples from 32 patients in several chromatin immunoprecipitation studies listed in Additional file 4: Table S3 and Additional file 6: Table S4. The tables in these Additional files include the following information for each patient: race, gender, age, diagnosis, ANCA subtype, disease status, BVAS, ANCA titer, serum creatinine, and treatment, WBC counts, and absolute neutrophil counts, if available.

### Microarray and quantitative RT-PCR

RNA from circulating leukocytes was extracted using RNA STAT-60 (Tel-Test “B”, Friendswood, TX, USA) and DNase treated. For the microarray study, 25 ANCA-patient samples were compared to 16 healthy controls by Affymetrix microarray gene chip for identification of gene expression levels, as previously described [13, 15, 48]. Differentially expressed genes were identified by analysis of variance (ANOVA) using Partek Genomics Suite (Partek GS 6.4, St Louis, CA) with a 1.2-fold change and *p* value <0.05 between patient and healthy control groups. Differentially expressed genes were analyzed using the Ingenuity Pathway Analysis (IPA; Java version 1.7.0_25 and 1.6.0_51; Ingenuity Systems, Redwood City, CA) to evaluate biological themes [49].

For quantitative RT-PCR, leukocyte RNA was analyzed from 40 PR3-ANCA patients and 40 MPO-ANCA patients (each group of 40: remission (BVAS = 0), *n* = 20; active (BVAS ≥ 3), *n* = 20), and 20 healthy controls. Quantitative detection of *MPO* and *PRTN3* mRNA levels from patient samples was determined using a standard curve. The standard curve for *MPO* mRNA levels was generated using HL60 cells, a cell positive for *MPO* mRNA, diluted with Jurkat cells, a cell line negative for *MPO* mRNA. The standard curve for *PRTN3* mRNA levels was generated using THP-1 cells, a cell positive for *PTRN3* mRNA, diluted with Jurkat cells, a cell line negative for *PRTN3* mRNA. MPO and PRTN3 mRNA levels for patients and healthy donor samples were determined by 2^−ΔΔCt^ calculations and expressed relative to standard curves. Primers and probes for *MPO* and *PRTN3* were previously published, and Cytochrome c oxidase (*COX5B*) was used as mRNA internal control [13]. Quantitative detection of *EHMT1/GLP*, *EHMT2/G9a*, *MSL1*, and *ING4* mRNA levels was determined by 2^−ΔΔCt^ calculations, with *COX5B* as the mRNA internal control, and expressed as fold change of reference control samples. Primers and probes were purchased from Applied Biosystems (Applied Biosystems, Foster City, CA). Quantitative RT-PCR assays were performed on an ABI PRISM 7900HT sequence detection system (Applied Biosystems), using the TaqMan EZ RT-PCR kit (Applied Biosystems).

### Monocyte and neutrophil isolation

Peripheral blood was collected in two 10 ml sodium heparin tubes (14–18 ml of blood). Red blood cells were depleted with 1 volume HetaSep (Stem Cell Technologies) for 5 volumes whole blood and centrifuged at 92*g*, 6 min, no brake. The nucleated cells were layered on Histopaque 1077 (Sigma) and centrifuged (400*g*, 30 min, no brake). PBMCs were washed once with PBS before isolating cell types using magnetic microbeads. CD14+ monocytes were isolated with the Human CD14 Positive Selection Kit as previously described (EasySep™, Stem Cell Technologies) [50, 51]. RNA was extracted from monocytes with the Qiagen AllPrep DNA/RNA Mini Kit (Qiagen, Chatsworth, CA). In parallel, neutrophils were isolated from the red blood cell/granulocyte pellet remaining after the Histopaque spin. The neutrophil pellet was washed once with PBS, and red cells were lysed with 5 ml water followed by 5 ml 2× PBS. Neutrophils were pelleted (300*g*, 10 min), lysed with STAT-60 (Tel-Test “B”, Friendswood, TX, USA), and RNA was extracted.

### Chromatin immunoprecipitation (ChIP)

ChIP for H3K9me2 was previously described [15]. ChIP for H4K16ac and H3K4me2 followed a similar procedure with the following modifications: neutrophils were isolated by HetaSep (StemCell Technologies, Vancouver, BC, Canada). Cells (5 × 10^6^ cells/ml/sample) were incubated with formaldehyde at a final concentration of 0.6 % to crosslink DNA and proteins, and then lysed and sonicated in lysis buffer without SDS (10 mM Tris-HCl, pH 8.0; 100 mM NaCl; 1 mM EDTA, pH 8.0; 0.5 mM EGTA; 0.1 % Na-Deoxycholate; 0.5 % *N*-Lauroylsarcosine). After sonication, lysate was diluted 1:1 in lysis buffer and Triton X-100 was added to 1 % final concentration. Anti-H3K9me2 antibody was purchased from Abcam (Abcam, Cambridge, MA), and anti-H3K4me2, H4K16ac, and H3K9,14ac antibodies from Millipore (EMD Millipore, Billerica, MA). Primers for Q-PCR of *PRTN3* and *MPO* promoter regions and control *MYO-D* were previously published [15]. *FCGR3B* promoter, sense primer (CCACCATAGAACAGGAATAG) and antisense primer (AGACCTTTGGGAGAGTAAA) were from Integrated DNA Technologies (Integrated DNA Technologies, Coralville, IA). Specific DNA was analyzed by quantitative PCR and expressed as a relative percent of input chromatin. The level of enrichment for the indicated histone modification was calculated using raw Ct values from qPCR of diluted input sample.

### Statistics

Descriptive statistics include mean and standard deviation, or median and interquartile range for non-normal distributions. Differences between two groups were compared by Wilcoxon test. Spearman rank correlations were used. Analyses and plots were conducted with SAS software (Version 9.3) (SAS Institute, Cary, NC). Microarray data was analyzed by ANOVA using Partek GS 6.4. Dot plots and box and whisker plots in each figure were generated by Partek GS 6.4.

## Additional files

Additional file 1: Table S1. Characteristics of patients with ANCA disease used to measure expression by microarray. (PDF 42.7 kb)
Additional file 2: Table S2. Characteristics of patients with ANCA disease used to measure expression by TaqMan qRT-PCR. (PDF 65.3 kb)
Additional file 3: Figure S1. Comparison of therapy in patients during remission on expression of histone modifying genes, EHMT1, EHMT2, ING4, and MSL1. (PDF 93.6 kb)
Additional file 4: Table S3. Characteristics of patients with ANCA disease used for chromatin immunoprecipitation. (PDF 46.2 kb)
Additional file 5: Figure S2. ChIP for active histone modification H3K9,14 in neutrophils and cell lines. (PDF 167 kb)
Additional file 6: Table S4. Histone modifications profiled by chromatin immunoprecipitation for each patient. (PDF 41.9 kb)
