# Supplementary material for: Histone modification signature at myeloperoxidase and proteinase 3 in patients with anti-neutrophil cytoplasmic autoantibody-associated vasculitis
Source: Clin Epigenetics. 2016 Aug 12;8:85. doi: 10.1186/s13148-016-0251-0 (PMC5057507; doi:10.1186/s13148-016-0251-0)
Supplement: Additional file 1: Table S1. — Characteristics of patients with ANCA disease used to measure expression by microarray. (PDF 42.7 kb) [file 13148_2016_251_MOESM1_ESM.pdf]

Additional file 1: Table S1. Characteristics of patients with ANCA disease used to measure expression by microarray

Patients	Race	Gender	Age	Diagnosis	ANCA subtype	Disease Status	BVAS	ANCA Titer		Serum Creatinine (mg/dl)	treatment	WBC x109th/L	absNeuts x109th/L
								PR3	MPO				
MA01	W	M	76	MPA	MPO-ANCA	ongoing	5	3.4	10.4	1.4	-	8.1	5.8
MA02	W	F	26	MPA	MPO-ANCA	remission	0	2.4	86.9	1.0	MMF	5.7	3.6
MA03	W	M	55	GPA	PR3-ANCA	ongoing	6	35.0	5.1	1.2	CS+MMF	10.4	7.7
MA04	W	M	55	GPA	PR3-ANCA	ongoing	6	32.0	5.4	1.2	CS	9.4	7.4
MA05	W	M	56	GPA	PR3-ANCA	ongoing	4	64.5	5.4	1.0	CS	12.4	11.1
MA06	W	F	54	MPA	MPO-ANCA	relapse	4	13.2	70.5	1.4	Rit8m	x	x
MA07	W	F	54	MPA	MPO-ANCA	ongoing	6	4.3	40.5	1.7	CS+CyA+Rit2m	13.5	x
MA08	B	M	46	GPA	PR3-ANCA	relapse	18	37.1	4.9	1.5	CS+MMF+PLEX	9.0	8.0
MA09	W	M	71	GPA	PR3-ANCA	remission	0	36.0	4.7	5.5	-	9.9	x
MA10	W	M	43	GPA	PR3-ANCA	first onset	15	13.5	3.5	2.1	CS+CP+PLEX	8.6	7.7
MA11	W	M	38	MPA	PR3-ANCA	ongoing	6	1230.0	3.5	4.0	MMF	x	x
MA12	W	M	64	MPA	MPO-ANCA	first onset	18	2.3	18.5	4.5	-	7.1	4.7
MA13	W	F	60	MPA	MPO-ANCA	relapse	6	2.4	40.5	0.9	PLEX	16.0	11.9
MA14	W	F	61	MPA	MPO-ANCA	ongoing	2	2.2	28.9	1.1	CS+CP	8.7	7.4
MA15	W	M	68	GPA	PR3-ANCA	ongoing	10	13.9	3.1	2.5	CS+CP	6.8	5.7
MA16	W	F	17	MPA	PR3-ANCA	ongoing	28	1669	2.9	0.9	CS	11.6	10.4
MA17	W	M	79	MPA	MPO-ANCA	first onset	16	3.4	100.3	9.0	CS+CP+PLEX+HD	12.2	10.8
MA18	B	F	51	GPA	PR3-ANCA	first onset	22	104.8	3.4	9.2	CS+PLEX+HD	x	14.6
MA19	W	F	45	MPA	MPO-ANCA	ongoing	1	3.9	43.3	1.2	CS+CP	6.4	5.0
MA20	W	M	72	Lim	PR3-ANCA	first onset	12	70.9	4.3	5.5	CS+HD	23.3	22.6
MA21	B	F	21	MPA	MPO-ANCA	first onset	5	3.6	57.6	1.0	CS	16.7	11.0
MA22	W	F	77	MPA	MPO-ANCA	first onset	21	2.8	107.4	5.0	CS+PLEX	10.4	10.0
MA23	W	F	65	MPA	PR3-ANCA	first onset	20	41.3	4.8	3.9	CS+AZ+PLEX	x	8.2
MA24	W	M	60	MPA	PR3-ANCA	first onset	21	100.7	4.0	3.2	CS+PLEX	21.3	17.4
MA25	B	F	28	MPA	MPO-ANCA	first onset	8	2.2	3.7	0.8	CS+CP	20.1	18.0

ANCA, antineutrophil cytoplasmic autoantibodies; PR3, proteinase 3; MPO, myeloperoxidase;
MPA, microscopic polyangiitis; GPA, granulomatosis with polyangiitis; CSS, Churg-Strauss Syndrome; Lim, renal-limited small vasculitis disease;
CS, corticosteroids; CP, cyclophosphamide; AZ, azathioprine; Cya, cyclosporin A; MMF, mycophenolate; Rit(x)m, months after rituximab;
PLEX, plasma exchange; HD, hemodialysis;
