# Supplementary material for: Histone modification signature at myeloperoxidase and proteinase 3 in patients with anti-neutrophil cytoplasmic autoantibody-associated vasculitis
Source: Clin Epigenetics. 2016 Aug 12;8:85. doi: 10.1186/s13148-016-0251-0 (PMC5057507; doi:10.1186/s13148-016-0251-0)
Supplement: Additional file 2: Table S2. — Characteristics of patients with ANCA disease used to measure expression by TaqMan qRT-PCR. (PDF 65.3 kb) [file 13148_2016_251_MOESM2_ESM.pdf]

Additional file 2: Table S2. Characteristics of patients with ANCA disease used to measure expression by TaqMan qRT-PCR

Patients	Race	Gender	Age	Diagnosis	ANCA Subtype	Disease Status	BVAS	ANCA Titer		Serum Creatinine (mg/dl)	Treatment	WBC $x10^9$th/L	absNeuts $x10^9$th/L
								PR3	MPO				
TM01	W	F	34	MPA	MPO-ANCA	remission	0	2.3	35.1	1.7	-	12.5	X
TM02	W	F	56	MPA	MPO-ANCA	remission	0	1.5	36.0	1.1	MMF	6.8	4.7
TM03	W	F	25	MPA	MPO-ANCA	remission	0	1.7	24.6	1.7	MMF	6.6	4.1
TM04	B	F	55	GPA	MPO-ANCA	remission	0	3.6	46.8	1.1	MMF	5.6	3.7
TM05	W	M	72	MPA	MPO-ANCA	remission	0	3.2	66.1	1.0	-	7.0	5.0
TM06	W	F	25	MPA	MPO-ANCA	remission	0	1.6	14.9	0.9	-	6.0	3.7
TM07	W	M	77	GPA	MPO-ANCA	remission	0	3.5	35.1	1.3	Rit7m	10.9	8.7
TM08	W	M	68	MPA	MPO-ANCA	remission	0	2.1	23.5	2.0	MMF	5.4	4.0
TM09	O	F	52	MPA	MPO-ANCA	remission	0	2.1	51.1	1.1	AZ	6.1	4.8
TM10	W	F	56	MPA	MPO-ANCA	remission	0	2.5	30.6	2.0	CS+MMF+Rit3m	9.3	7.8
TM11	W	F	57	Lim	MPO-ANCA	remission	0	2.2	13.4	1.0	AZ+Rit1m	X	X
TM12	H	F	10	MPA	MPO-ANCA	remission	0	2.6	4.1	0.8	CS+AZ+Rit3m	6.5	3.6
TM13	B	F	22	MPA	MPO-ANCA	remission	0	2.5	12.1	17.5	HD	4.7	3.1
TM14	H	M	47	MPA	MPO-ANCA	remission	0	2.5	5.1	1.1	-	6.8	X
TM15	W	F	53	MPA	MPO-ANCA	remission	0	3.6	7.1	1.0	-	5.2	2.8
TM16	W	F	61	MPA	MPO-ANCA	remission	0	2.1	6.9	1.4	-	5.9	4.4
TM17	W	F	54	Lim	MPO-ANCA	remission	0	3.1	59.2	1.3	CS+AZ	5.8	4.5
TM18	H	F	48	GPA	MPO-ANCA	remission	0	1.8	12.7	2.0	AZ	6.8	X
TM19	B	F	85	MPA	MPO-ANCA	remission	0	2.0	3.7	2.1	AZ	5	3.6
TM20	B	F	57	Lim	MPO-ANCA	remission	0	2.2	2.4	1.6	CS+Rit6m	2.7	1.6
TM21	W	M	76	MPA	MPO-ANCA	ongoing	5	3.4	10.4	1.4	-	8.1	5.8
TM22	W	M	79	Lim	MPO-ANCA	first onset	12	5.0	25.0	3.5	CS+CP	14.7	X
TM23	W	M	55	MPA	MPO-ANCA	first onset	17	3.4	72.3	2.6	CS+CP	13.6	10.0
TM24	W	F	59	MPA	MPO-ANCA	relapse	16	2.4	40.5	0.8	CS+PLEX+CP	20.7	17.6
TM25	W	M	78	MPA	MPO-ANCA	first onset	11	3.4	100.3	3.3	CS+PLEX+HD	9.3	8.3
TM26	W	F	45	MPA	MPO-ANCA	first onset	15	12.3	102.4	1.0	CS+PLEX	14.1	X
TM27	W	F	77	MPA	MPO-ANCA	first onset	21	2.8	107.4	4.2	CS+PLEX	11.9	10.0
TM28	W	M	62	MPA	MPO-ANCA	first onset	18	5.9	81.4	7.1	CS	10.3	8.8
TM29	W	M	62	EGPA	MPO-ANCA	ongoing	24	5.9	21.5	0.9	CS	13.2	11.7
TM30	W	M	23	MPA	MPO-ANCA	first onset	20	5.6	69.9	2.2	CS	16.3	X
TM31	W	F	63	GPA	MPO-ANCA	ongoing	9	2.9	49.2	1.4	CS+CP	7.1	6.3
TM32	O	F	87	MPA	MPO-ANCA	relapse	10	1.9	39.4	1.1	AZ	8.3	6.3
TM33	W	M	61	Lim	MPO-ANCA	ongoing	5	1.9	55.9	1.3	CS+CP	11.3	10.6
TM34	W	F	73	GPA	MPO-ANCA	first onset	14	2.6	84.1	4.7	CS+HD	12.1	X
TM35	W	M	82	Lim	MPO-ANCA	ongoing	6	1.9	72.0	2.8	CS+CP	9.3	8.2
TM36	W	M	73	Lim	MPO-ANCA	first onset	16	1.9	80.3	5.8	CS+CP	11.8	10.1
TM37	B	M	65	Lim	MPO-ANCA	first onset	14	3.1	101.1	6.2	CS+PLEX	20.3	X
TM38	B	M	20	EGPA	MPO-ANCA	ongoing	3	2.2	44.8	1.9	CS+MMP+Rit0m	9.5	6.0
TM39	W	M	17	MPA	MPO-ANCA	ongoing	6	1.4	2.4	1.4	CS+CP+Rit2m	6.4	4.6
TM40	W	M	74	Lim	MPO-ANCA	ongoing	12	3.3	39.7	3.1	CS+CP	7.6	7.1
TM41	W	M	55	GPA	PR3-ANCA	remission	0	21.9	1.5	1.4	MMF	X	X
TM42	W	F	51	MPA	PR3-ANCA	remission	0	97.3	2.6	0.9	-	6.4	X
TM43	W	F	55	GPA	PR3-ANCA	remission	0	8.9	2.5	0.7	CS+Rit5m	9.0	7.2
TM44	W	F	46	GPA	PR3-ANCA	remission	0	84.9	2.7	1.0	MMF+Rit7m	X	X
TM45	W	F	25	MPA	PR3-ANCA	remission	0	28.7	2.4	0.9	-	6.0	2.6

TM46	W	F	82	MPA	PR3-ANCA	remission	0	3.3	1.9	1.3	-	7.8	3.9
TM47	W	M	55	GPA	PR3-ANCA	remission	0	18.4	1.9	1.3	MMF+Rit4m	3.9	2.0
TM48	W	M	63	MPA	PR3-ANCA	remission	0	76.4	2.8	2.1	CS	7.8	6.8
TM49	O	F	59	MPA	PR3-ANCA	remission	0	20.7	2.3	0.9	AZ	6.0	3.8
TM50	W	M	49	GPA	PR3-ANCA	remission	0	74.0	4.0	1.0	CS+MMF	6.3	4.9
TM51	B	F	56	MPA	PR3-ANCA	remission	0	93.6	1.9	0.8	MMF+Rit1m	5.9	3.8
TM52	W	M	62	MPA	PR3-ANCA	remission	0	4.3	3.2	1.4	-	6.1	X
TM53	W	F	63	MPA	PR3-ANCA	remission	0	55.3	3.6	1.0	MMF	4.0	X
TM54	W	F	75	GPA	PR3-ANCA	remission	0	2.6	4.6	1.0	AZ	6.7	4.7
TM55	W	M	54	MPA	PR3-ANCA	remission	0	31.4	2.2	3.1	Rit6m	6.2	3.8
TM56	B	F	55	MPA	PR3-ANCA	remission	0	8.5	1.9	0.8	MMF	4.1	2.4
TM57	W	F	18	GPA	PR3-ANCA	remission	0	10.4	2.3	0.9	CS+AZ+Rit10m	X	X
TM58	W	F	33	GPA	PR3-ANCA	remission	0	95.3	2.1	1.6	Rit9m	8.4	X
TM59	W	F	31	GPA	PR3-ANCA	remission	0	12.8	2.9	x	AZ	X	X
TM60	W	F	62	MPA	PR3-ANCA	remission	0	63.0	1.4	1.3	Rit6m	6.6	X
TM61	W	M	43	GPA	PR3-ANCA	ongoing	10	13.5	3.5	1.9	CS+CP+PLEX	12.4	X
TM62	W	M	67	GPA	PR3-ANCA	ongoing	10	13.9	3.1	2.5	CS+CP	6.8	5.7
TM63	W	F	17	MPA	PR3-ANCA	ongoing	28	1669.0	2.9	0.9	CS	11.6	10.4
TM64	B	F	51	GPA	PR3-ANCA	first onset	22	104.8	3.4	9.2	CS+PLEX+HD	16	14.6
TM65	W	M	22	MPA	PR3-ANCA	ongoing	5	26.5	5.1	2.6	CS+ME	7.4	6.7
TM66	W	M	60	MPA	PR3-ANCA	first onset	21	100.7	4.0	3.2	CS+PLEX	18.8	17.4
TM67	W	M	34	PMA	PR3-ANCA	first onset	18	1181.0	5.2	2.3	CS+PLEX	29.0	20.9
TM68	W	F	61	GPA	PR3-ANCA	relapse	12	92.3	4.7	1.1	CS+CP	12.5	11.4
TM69	W	F	65	MPA	PR3-ANCA	relapse	3	14.5	1.2	1.1	CS+Rit0m	X	X
TM70	O	F	24	GPA	PR3-ANCA	ongoing	6	170.0	3.7	x	CS	X	X
TM71	W	M	20	GPA	PR3-ANCA	relapse	6	2.3	1.7	1.2	CS+AZ	13.5	11.8
TM72	W	M	62	GPA	PR3-ANCA	ongoing	6	38.8	3.3	1.8	CP	8.3	4.2
TM73	W	M	57	GPA	PR3-ANCA	relapse	19	111.1	4.0	1.2	CP+ME	12.6	11.0
TM74	W	F	70	MPA	PR3-ANCA	first onset	9	126.0	4.8	1.0	CS	15.2	X
TM75	W	M	35	GPA	PR3-ANCA	ongoing	12	143.1	2.0	0.7	CS+CP	15.8	X
TM76	W	M	34	MPA	PR3-ANCA	first onset	20	>200	3.0	6.2	CS+CP+PLEX+HD	9.7	X
TM77	W	M	48	GPA	PR3-ANCA	ongoing	3	55.4	2.5	6.2	CS+CP+Rit4m	16.8	11.0
TM78	W	M	36	GPA	PR3-ANCA	first onset	20	92.1	2.4	2.2	CS+PLEX	26.1	24.6
TM79	W	F	23	GPA	PR3-ANCA	ongoing	23	114.8	3.8	1.2	CS+AZ	13.5	12.6
TM80	W	M	69	GPA	PR3-ANCA	ongoing	13	159.5	2.3	2.7	CS+CP	14.4	13.1
TM81	W	F	59	GPA	MPO-ANCA	ongoing	3	3.9	21.3	0.71	CS	14.0	12.5
TM82	B	M	57	MPA	MPO-ANCA	first onset	20	3.0	140.1	1.35	CS+PLEX+Rit1d	15.4	X
TM83	W	M	33	MPA	MPO-ANCA	first onset	8	3.1	6.4	0.81	CS+Rit2w+AZA	X	X
TM84	W	M	80	MPA	MPO-ANCA	relapse	8	1.3	11.5	2.30	CS+AZA	X	X
TM85	W	F	62	LIM	MPO-ANCA	first onset	18	2.8	63.0	4.74	CS+CP+Rit2w	8.4	6.5
TM86	W	M	69	MPA	MPO-ANCA	first onset	12	1.8	108.0	3.18	-	9.6	7.0
TM87	W	F	70	GPA	MPO-ANCA	relapse	6	1.5	151.4	1.24	CS+MTX	15.0	13.2
TM88	U	F	50	GPA	MPO-ANCA	relapse	6	5.1	118.5	1.50	CS+AZA	9.8	8.9
TM89	W	M	83	Lim	MPO-ANCA	relapse	15	1.7	51.5	3.26	-	8.8	X
TM90	W	M	51	GPA	PR3-ANCA	first onset	7	110.8	1.4	0.64	CS	26.6	X
TM91	W	M	76	GPA	PR3-ANCA	relapse	22	200.0	1.6	6.54	CS+PLEX+HD	11.4	X
TM92	W	M	67	MPA	PR3-ANCA	ongoing	1	27.7	1.7	1.33	Rit2w	8.6	5.6
TM93	W	M	62	GPA	PR3-ANCA	ongoing	6	22.1	1.5	1.06	CS+CP	8.4	6.3

note: patient idenitfication numbers marked with an asterix (*) were also used in the ChIP for histone modifications cohort

ANCA, antineutrophil cytoplasmic autoantibodies; PR3, proteinase 3; MPO, myeloperoxidase;
MPA, microscopic polyangiitis; GPA, granulomatosis with polyangiitis; CSS, Churg-Strauss Syndrome; Lim, renal-limited small vasculitis disease;
CS, corticosteroids; CP, cyclophosphamide; AZ, azathioprine; Cya, cyclosporin A; MMF, mycophenolate; Rit(x)m,w, or d months, weeks, or days after rituximab;
PLEX, plasma exchange; HD, hemodialysis;
TaqMan qRT-PCR was performed on RNA from total leukocytes of patients TM01-TM80; TaqMan qRT-PCR was performed on RNA from monocytes and PMNs of patients TM81-TM93
