# Supplementary material for: Histone modification signature at myeloperoxidase and proteinase 3 in patients with anti-neutrophil cytoplasmic autoantibody-associated vasculitis
Source: Clin Epigenetics. 2016 Aug 12;8:85. doi: 10.1186/s13148-016-0251-0 (PMC5057507; doi:10.1186/s13148-016-0251-0)
Supplement: Additional file 3: Figure S1. — Comparison of therapy in patients during remission on expression of histone modifying genes, EHMT1, EHMT2, ING4, and MSL1. (PDF 93.6 kb) [file 13148_2016_251_MOESM3_ESM.pdf]

Additional File 3: Figure S1

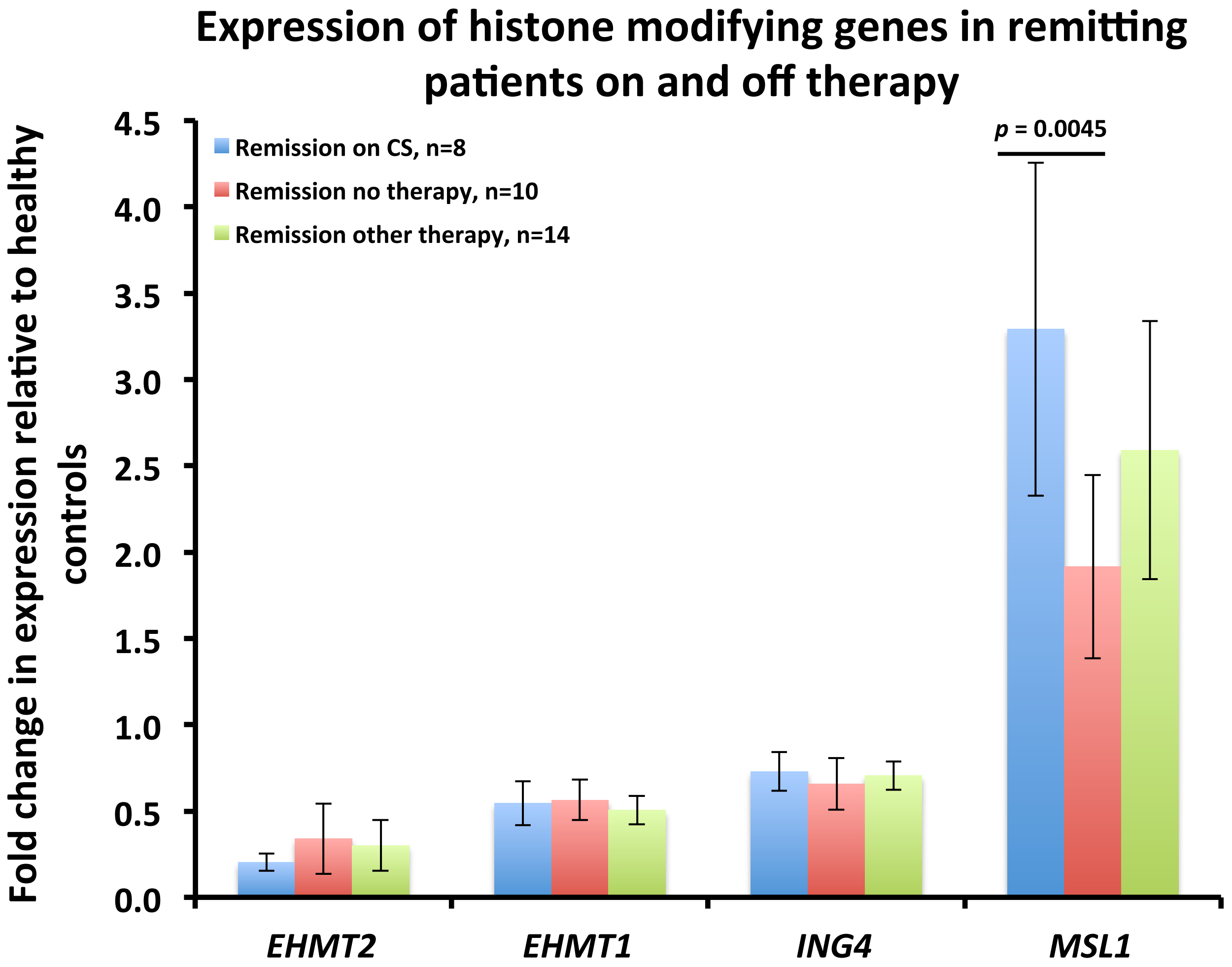

Additional File 3: Figure S1. Comparison of therapy in patients during remission on expression of histone modifying genes, *EHMT1*, *EHMT2*, *ING4*, and *MSL1*. Bars depict the average fold change in expression compared to healthy controls for patients in remission receiving corticosteroids (CS) (blue), patients in remission on no therapy (red), and patients in remission receiving therapy other than corticosteroids (green). See Additional file 2: Table S2 for a list of therapies other than corticosteroids. Error bars represent the standard deviation. Pairwise comparisons were performed using a two-tailed T-test, which demonstrated only the expression of *MSL1* was significantly different between remission patients on corticosteriods compared to remission patients on no therapy.
