# Supplementary material for: Histone modification signature at myeloperoxidase and proteinase 3 in patients with anti-neutrophil cytoplasmic autoantibody-associated vasculitis
Source: Clin Epigenetics. 2016 Aug 12;8:85. doi: 10.1186/s13148-016-0251-0 (PMC5057507; doi:10.1186/s13148-016-0251-0)
Supplement: Additional file 4: Table S3. — Characteristics of patients with ANCA disease used for chromatin immunoprecipitation. (PDF 46.2 kb) [file 13148_2016_251_MOESM4_ESM.pdf]

Additional file 4: Table S3. Characteristics of patients with ANCA disease used for chromatin immunoprecipitation

Patients	Race	Gender	Age	Diagnosis	ANCA Subtype	Disease Status	BVAS	ANCA Titer		Serum Creatinine (mg/dl)	Treatment	WBC x10^{9}th/L	absNeuts x10^{9}th/L
								PR3	MPO				
CH01	W	F	31	MPA	MPO-ANCA	Remission	0	2.8	69.4	1.7	-	8.5	5.8
CH02	W	M	32	GPA	PR3-ANCA	Ongoing	1	89.7	2.5	1.2	-	6.1	3.7
CH03	W	F	49	MPA	PR3-ANCA	Relapse	7	95.9	3.0	0.8	MMF+Ritl0m	7.3	5.9
CH04	W	F	43	GPA	PR3-ANCA	Relapse	6	11.8	1.3	0.8	CS+MMF	12.5	11.5
CH05	W	M	76	GPA	MPO-ANCA	Remission	0	3.5	56.4	x	MMF	X	X
CH06*	W	F	65	MPA	PR3-ANCA	Relapse	3	14.5	1.2	1.1	CS+Rit1m	x	x
CH07	W	M	51	GPA	PR3-ANCA	Ongoing	8	65.3	3.5	1.5	CS+MMF	X	x
CH08	B	F	63	GPA	MPO-ANCA	Ongoing	5	6.8	34.1	2.2	CS	4.6	2.9
CH09	O	F	83	MPA	MPO-ANCA	Relapse	16	2.3	60.1	1.7	CS	13.5	12.5
CH10	W	M	59	GPA	PR3-ANCA	Ongoing	5	29.7	2.4	2.9	CS+CP+Rit1m	9.2	8.8
CH11	W	M	49	GPA	PR3-ANCA	New onset	19	106.6	2.5	7.4	CS+PLEX+HD	X	X
CH12	O	M	40	GPA	MPO-ANCA	Ongoing	3	3.6	34.6	1.1	CS+AZ	6.4	4.5
CH13	B	F	29	Lim	MPO-ANCA	Unclear - there is no note	0	3.0	8.4	1.0	CS+CP	12.6	10.5
CH14	W	M	72	MPA	MPO-ANCA	New onset	16	3.8	122.7	2.8	CS+PLEX	15.1	14.1
CH15	W	M	59	GPA	PR3-ANCA	New onset	12	198.8	3.9	0.8	CS	18.6	14.4
CH16	W	M	28	MPA	PR3-ANCA	New onset	25	136.6	4.2	5.0	CS+CP+PLEX	23.7	20.8
CH17	W	M	63	MPA	MPO-ANCA	Remission	0	2.3	22.7	2.2	CS+CP	9.3	8.7
CH18	W	F	19	GPA	PR3-ANCA	New onset	24	91.3	2.1	1.5	CS	23	21.2
CH19	W	F	51	MPA	MPO-ANCA	New onset	16	2.2	17.7	0.7	CS	5.5	3
CH20	W	F	59	GPA	PR3-ANCA	Relapse	10	101.0	1.0	1.5	CS	10.1	8.3
CH21*	B	M	20	EGPA	MPO-ANCA	ongoing	3	2.2	44.8	1.9	CS+MMP+Ritl0m	9.5	6
CH22*	W	M	35	GPA	PR3-ANCA	ongoing	12	143.1	2.0	0.7	CS+CP	15.8	X
CH23	W	M	65	MPA	PR3-ANCA	ongoing	6	81.5	2.0	1.5	Rit11m	7.7	4.8
CH24	B	M	58	MPA	MPO-ANCA	Remission	0	2.3	4.4	2.9	CS+CP	12.2	9.3
CH25	W	M	79	Lim	MPO-ANCA	Remission	0	2.2	23.6	2.0	-	X	X
CH26*	W	M	48	GPA	PR3-ANCA	ongoing	3	55.4	2.5	6.2	CS+CP+Rit4m	16.8	11
CH27*	W	M	69	GPA	PR3-ANCA	ongoing	13	159.5	2.3	2.7	CS+CP	14.4	13.1
CH28	W	F	30	MPA	PR3-ANCA	Relapse	10	31.7	2.2	1.7	AZ+Rit12m	5.1	X
CH29*	W	M	74	Lim	MPO-ANCA	ongoing	12	3.3	39.7	3.1	CS+CP	7.6	7.1
CH30	W	F	71	MPA	ANCA Neg	Remission	0	1.9	1.6	0.9	CS+Rit	5.8	X
CH31	W	F	19	MPA	MPO-ANCA	ongoing	5	2.7	6.5	0.5	CS	12.3	11.4
CH32	W	M	65	Lim	ANCA Neg	New onset	12	2.8	1.9	5.5	CS+PLEX+HD	6.4	5.6

note: patient idenitfication numbers marked with an asterix (*) were also used in the qRT-PCR cohort
ANCA, antineutrophil cytoplasmic autoantibodies; PR3, proteinase 3; MPO, myeloperoxidase;
MPA, microscopic polyangiitis; GPA, granulomatosis with polyangiitis; CSS, Churg-Strauss Syndrome; Lim, renal-limited small vasculitis disease;
CS, corticosteroids; CP, cyclophosphamide; AZ, azathioprine; Cya, cyclosporin A; MMF, mycophenolate; Rit(x)m, months after rituximab;
PLEX, plasma exchange; HD, hemodialysis;
