# Supplementary material for: Histone modification signature at myeloperoxidase and proteinase 3 in patients with anti-neutrophil cytoplasmic autoantibody-associated vasculitis
Source: Clin Epigenetics. 2016 Aug 12;8:85. doi: 10.1186/s13148-016-0251-0 (PMC5057507; doi:10.1186/s13148-016-0251-0)
Supplement: Additional file 5: Figure S2. — ChIP for active histone modification H3K9,14 in neutrophils and cell lines. (PDF 167 kb) [file 13148_2016_251_MOESM5_ESM.pdf]

Additional file 5: Figure S2

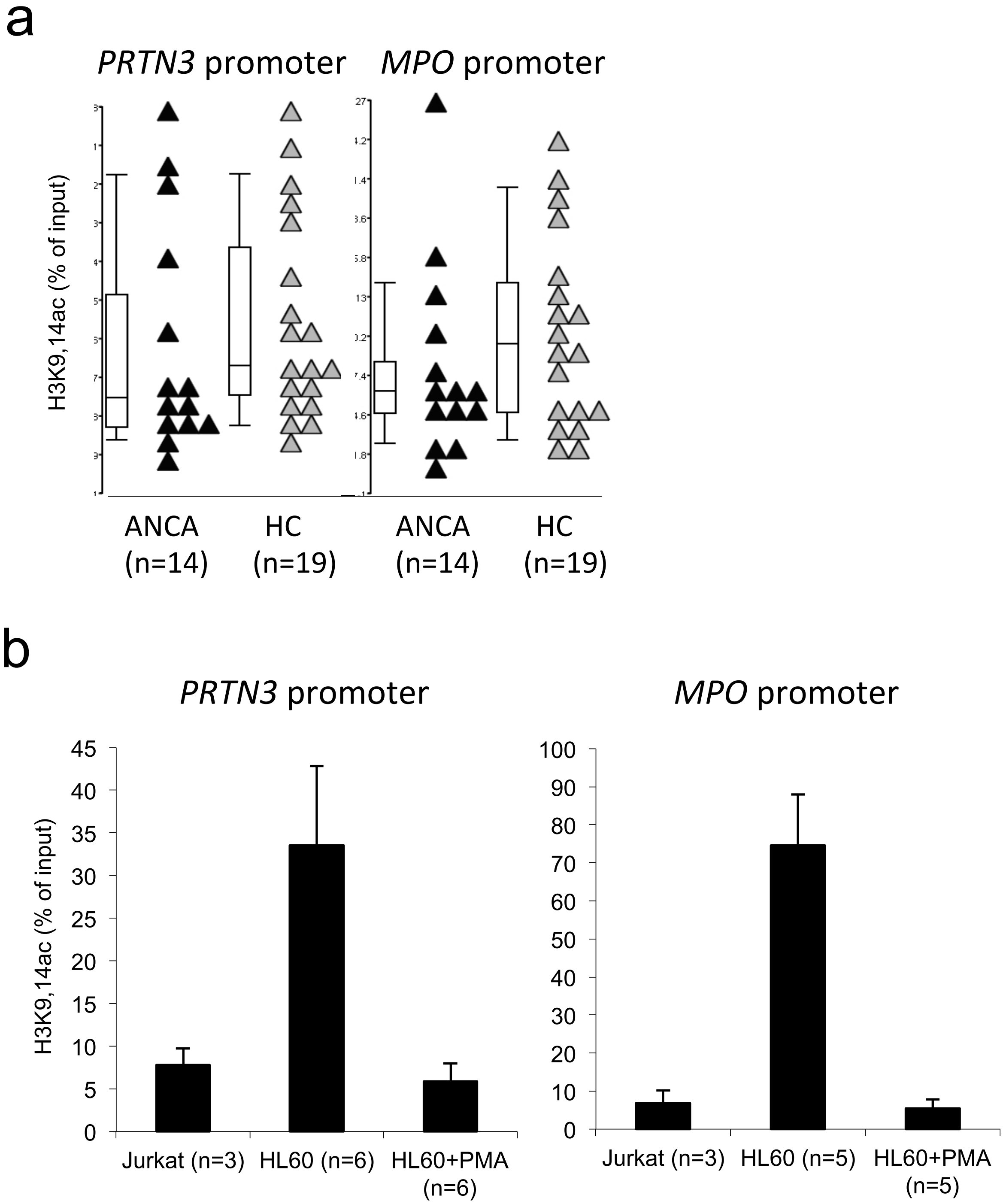

Additional file 5: Figure S2. ChIP for active histone modification H3K9,14 in neutrophils and cell lines. **a.** ChIP-quantitative PCR of neutrophils for the active histone modification H3K9,14 acetylation. At *PRTN3* and *MPO* promoters the level of H3K9,14ac is similar between ANCA patients and healthy controls (HC). **b.** ChIP-quantitative PCR for H3K9,14 acetylation in cell lines that do not express (Jurkat) or do express (HL60) *PRTN3* and *MPO*. ChIP for H3K9,14 was performed on HL60 cells treated with 100nM PMA for 24 hours which induces HL60 cells to differentiate and silence *PRTN3* and *MPO*. The level of acetylated H3K9,14 was calculated using raw Ct values from qPCR of diluted input.
