# Supplementary material for: Histone modification signature at myeloperoxidase and proteinase 3 in patients with anti-neutrophil cytoplasmic autoantibody-associated vasculitis
Source: Clin Epigenetics. 2016 Aug 12;8:85. doi: 10.1186/s13148-016-0251-0 (PMC5057507; doi:10.1186/s13148-016-0251-0)
Supplement: Additional file 6: Table S4. — Histone modifications profiled by chromatin immunoprecipitation for each patient. (PDF 41.9 kb) [file 13148_2016_251_MOESM6_ESM.pdf]

Additional file 6: Table S4. Histone modifications profiled by chromatin immunoprecipitation for each patient

Patients	me2 H3K9	me3 H3K27	ac H4K16	me2 H3K4	acH3 K9,14
CH01	√	√			√
CH02	√	√			√
CH03	√	√	√		√
CH04	√	√			√
CH05			√		
CH06*			√	√	
CH07	√	√			
CH08	√	√			
CH09	√	√			
CH10	√	√	√		
CH11	√	√	√		√
CH12	√	√	√		√
CH13			√		
CH14	√	√			√
CH15	√	√	√		√
CH16	√	√	√		√
CH17	√	√	√		√
CH18	√	√	√		√
CH19			√		
CH20			√		
CH21*			√	√	
CH22*			√	√	
CH23			√		
CH24			√	√	
CH25			√	√	
CH26*			√	√	
CH27*			√	√	
CH28			√		
CH29*			√	√	
CH30			√		
CH31			√		
CH32			√		

note: patient numbers marked with an asterix (*) were also used in the qRT-PCR cohort
